# Supplementary material for: The Effect of Meloxicam on Inflammatory Response and Oxidative Stress Induced by Klebsiella pneumoniae in Bovine Mammary Epithelial Cells
Source: Vet Sci. 2024 Nov 29;11(12):607. doi: 10.3390/vetsci11120607 (PMC11680352; doi:10.3390/vetsci11120607)
Supplement: Supplementary file 1 [file vetsci-11-00607-s001.zip › vetsci-3275568-supplementary.pdf]

The WB figure in repeat 1 is COX-1 and β-actin in manuscript Figure 3B

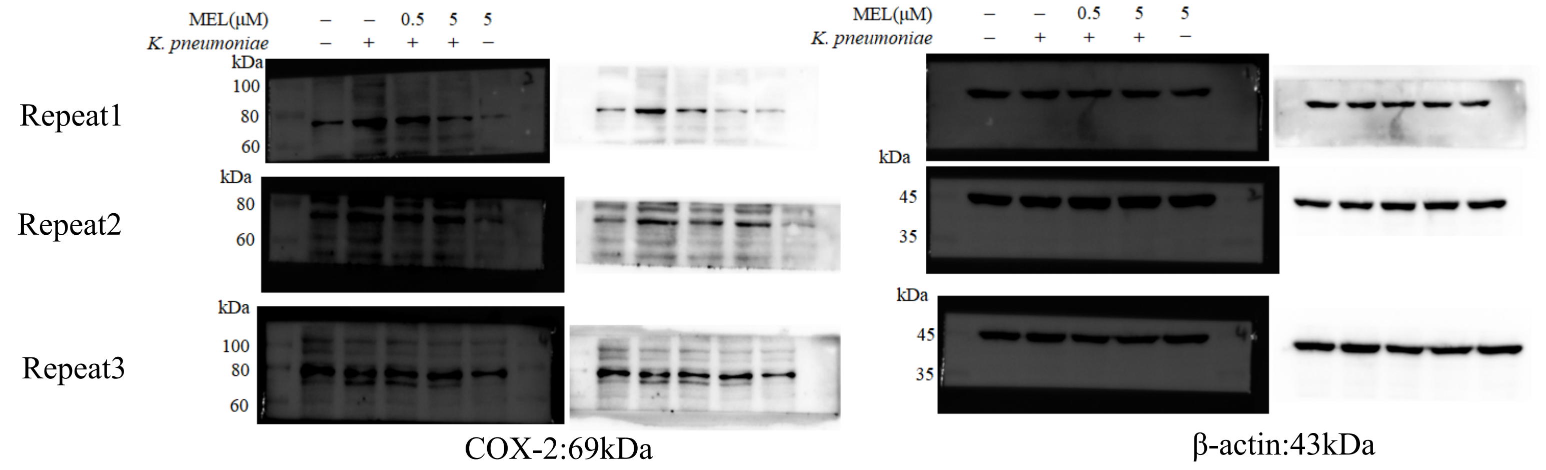

The WB figure in repeat 3 is p-p65, p65 and β-actin in manuscript Figure 5A

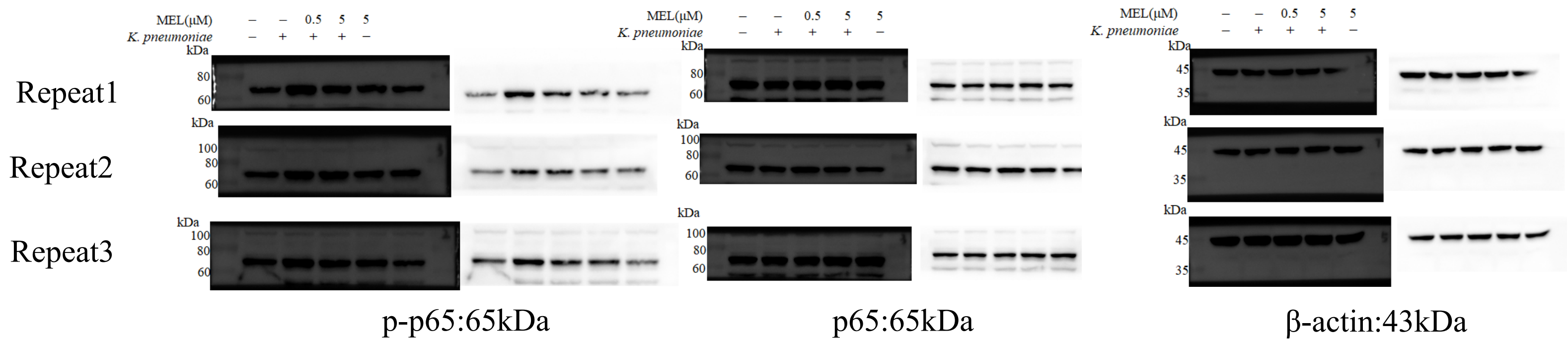

The WB figure in repeat 3 is p-IκB and IκB in manuscript Figure 5A

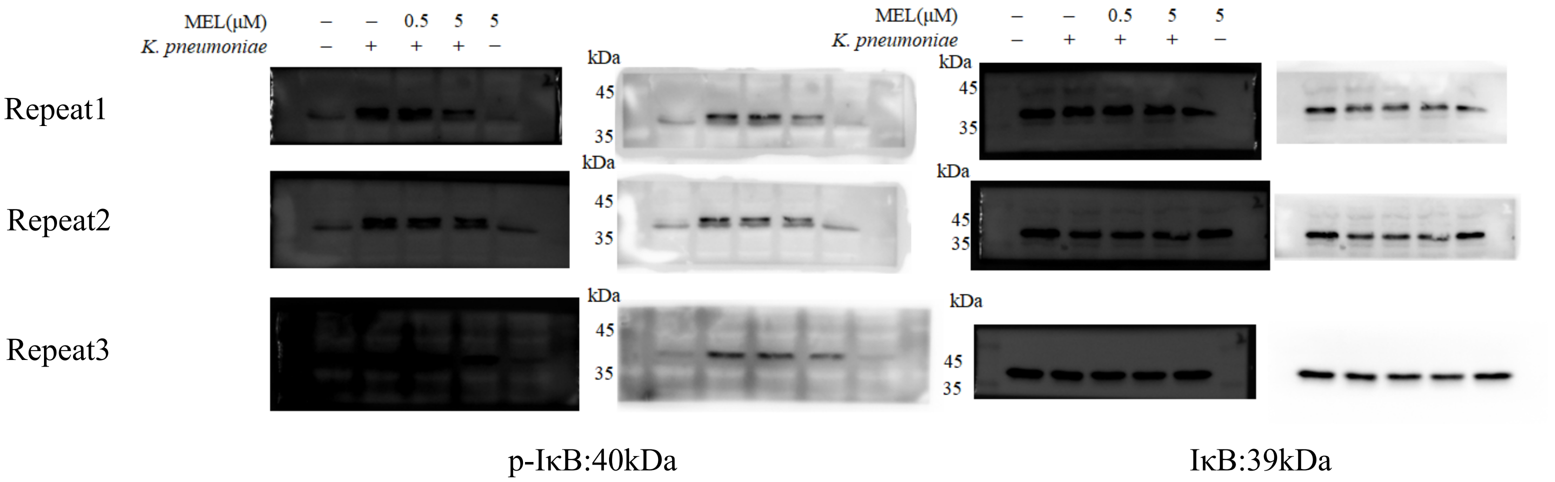

The WB figure in repeat 1 is Nrf2 in manuscript Figure 9A

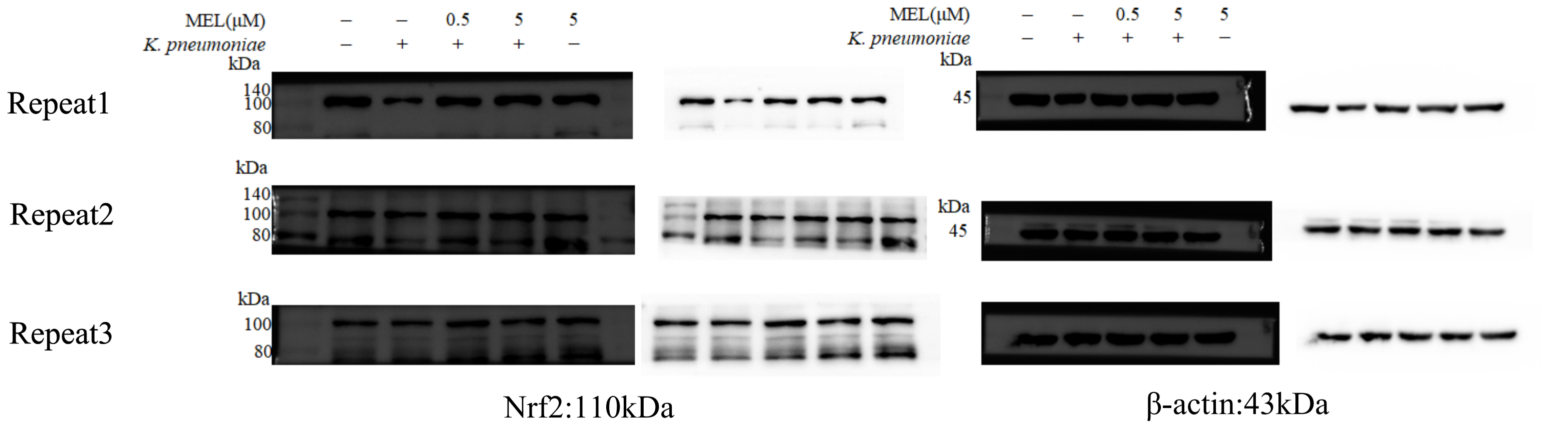

The WB figure in repeat 1 is β-actin in manuscript Figure 9A; The WB figure in repeat 2 is Keap1 in manuscript Figure 9A

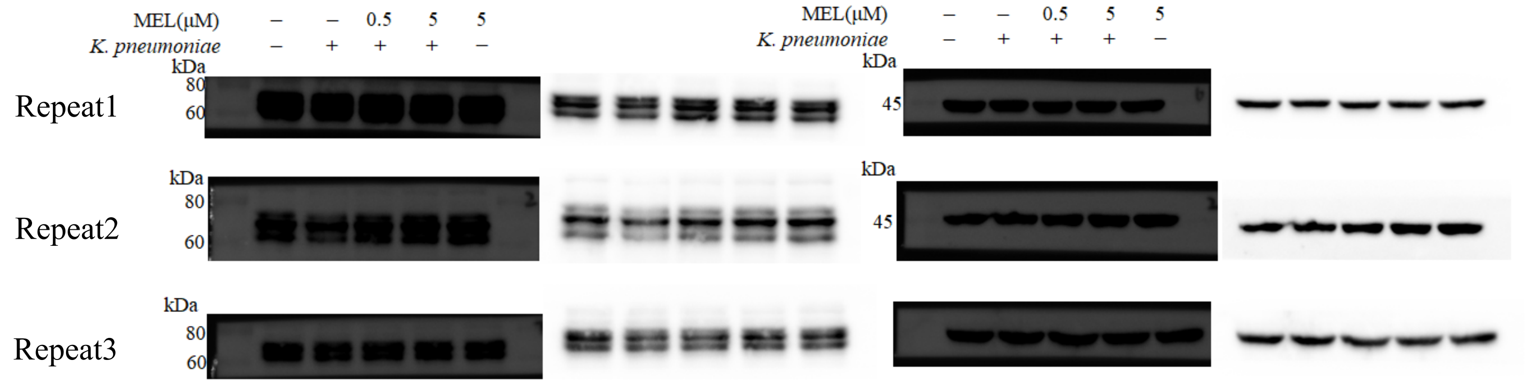

Keap1:70kDa β-actin:43kDa

The WB figure in repeat 2 is HO-1 in manuscript Figure 9A

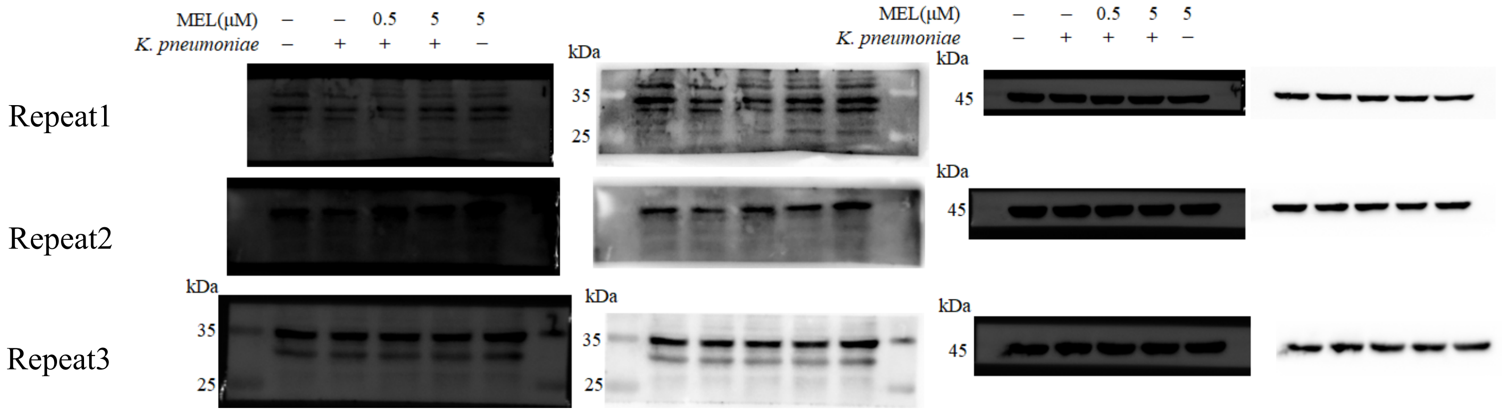

HO-1:33kDa β-actin:43kDa

The WB figure in repeat 3 is NQO-1 in manuscript Figure 9A

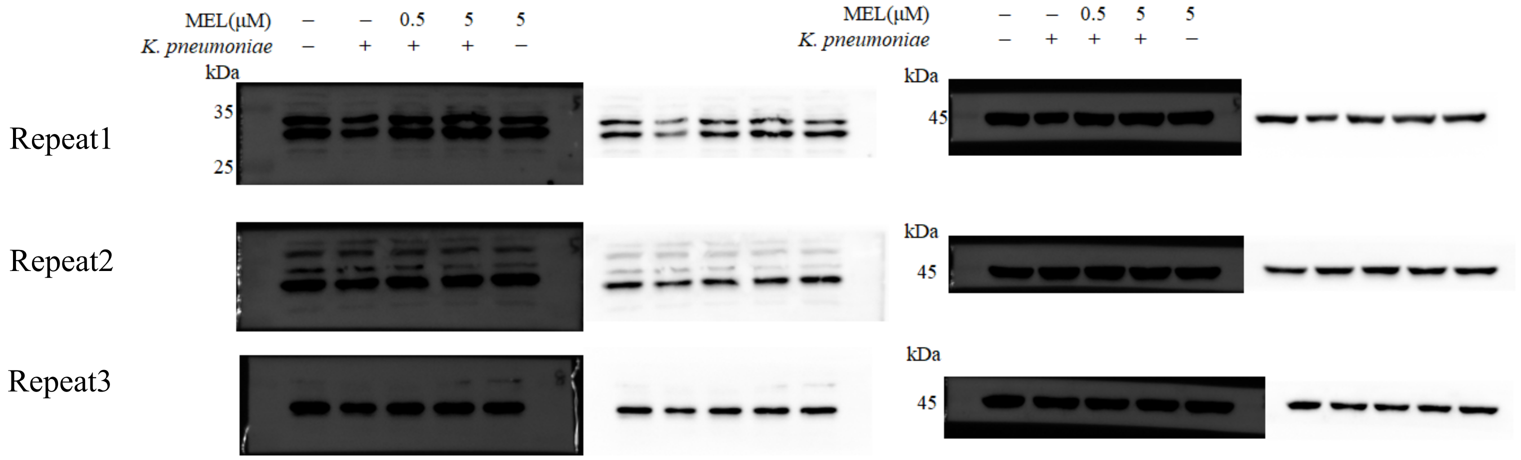

NQO-1:31kDa β-actin:43kDa

The WB figure in repeat 1 is n-Nrf2 and Lamin B1 in manuscript Figure 9A

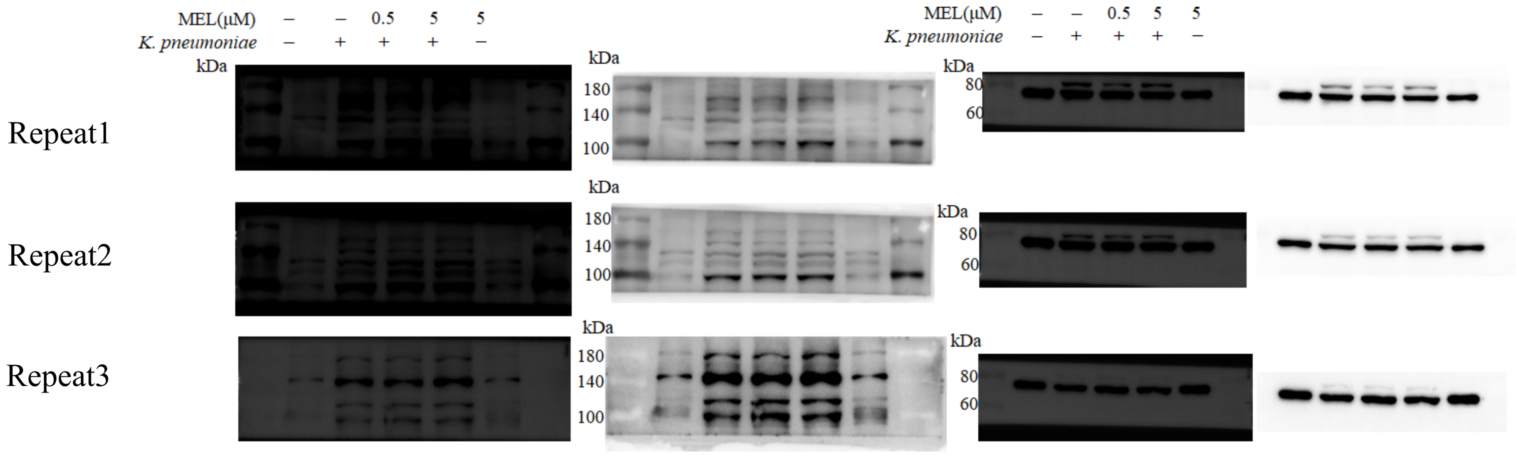

n-Nrf2:110kDa Lamin B1:66kDa
